# At-hatch administration of probiotic to chickens can introduce beneficial changes in gut microbiota

**DOI:** 10.1371/journal.pone.0194825

**Published:** 2018-03-23

**Authors:** Stephen Baldwin, Robert J. Hughes, Thi Thu Hao Van, Robert J. Moore, Dragana Stanley

**Affiliations:** 1 Central Queensland University, Institute for Future Farming Systems, Rockhampton, Queensland, Australia; 2 South Australian Research and Development Institute, Roseworthy, South Australia, Australia; 3 The University of Adelaide, School of Animal and Veterinary Sciences Roseworthy, South Australia, Australia; 4 RMIT University, School of Science, Bundoora, Victoria, Australia; Texas A&M University College Station, UNITED STATES

## Abstract

Recent advances in culture-free microbiological techniques bring new understanding of the role of intestinal microbiota in heath and performance. Intestinal microbial communities in chickens assume a near-stable state within the week which leaves a very small window for permanent microbiota remodelling. It is the first colonisers that determine the fate of microbial community in humans and birds alike, and after the microbiota has matured there are very small odds for permanent modification as stable community resists change. In this study we inoculated broiler chicks immediately post hatch, with 3 species of *Lactobacillus*, identified by sequencing of 16S rRNA and pheS genes as *L*. *ingluviei*, *L*. *agilis* and *L*. *reuteri*. The strains were isolated from the gut of healthy chickens as reproducibly persistent *Lactobacillus* strains among multiple flocks. Birds inoculated with the probiotic mix reached significantly higher weight by 28 days of age. Although each strain was able to colonise when administered alone, administering the probiotic mix at-hatch resulted in colonisation by only *L*. *ingluviei*. High initial abundance of *L*. *ingluviei* was slowly reducing, however, the effects of at-hatch administration of the *Lactobacillus* mix on modifying microbiota development and structure remained persistent. There was a tendency of promotion of beneficial and reduction in pathogenic taxa in the probiotic administered group.

## Introduction

The gastrointestinal tract (GIT) of broilers plays an important role in their health and performance [1]. GIT health is dependent upon complex interactions between diet, host bacterial community (microbiota) and intestinal functioning [2]. When gut health is compromised digestion and nutrient absorption are affected resulting in poor feed utilisation and susceptibility to disease [3]. Studies of broilers fed probiotics have demonstrated improved weight gain, and improved feed conversion ratios [4–8].

Recent studies have powerfully linked GIT microbiota to health in animals including chicken (reviewed in Stanley *et al*. [1]) and humans [9–11]. Other studies confirmed a link between GIT microbiota and obesity [12], suggesting that the manipulation of intestinal microbiota could be used to manipulate weight gain. Interactions between host and microbiota are, however, complex, and can have a positive or negative effect [13].

Microbes that confer health and performance benefits are called probiotics [14]. The mechanisms of probiotic action include: scavenging free-radicals [15], producing bacteriocins [16], influencing intestinal mucin gene expression [17], exclusion and inhibition of pathogens [18–20], and virulence attenuation [21]. Fuller [22] summarized the benefits as improved feed conversion rates, increased growth rates, greater disease resistance, improved digestion and absorption of nutrients, improved carcass quality and reduction of zoonotic bacteria. Sanders [23] reported a reduction in lactose intolerance and modulated immune system functions. According to Conway [24] and Dunne et al. [25], probiotic strains should be of host origin, non-pathogenic, suitable for manufacturing and delivery, capable of adhering to gut mucosa, modulate immune function, improve intestinal function and growth and produce non-toxic metabolites. The most reported health benefits of probiotics have been improvements in digestive health and function [26]. The GIT microbiota aids in digestion and absorption of nutrients by catabolizing substrates [27].

Probiotics are a promising alternative to antibiotics because they can control pathogens [28, 29]. Probiotics can competitively exclude pathogens in the intestine by successfully competing for nutrients, adhering to the mucosa, modulating a favorable immune response, secreting bacteriocins, creating a favorable environment (eg. lowering pH by secreting lactic and acetic acid), and regulating colonocyte gene expression [30–32]. Bacteria that lower the pH of the gut by secreting lactic acid (eg. *Lactobacillus*) are considered suitable candidate [33] for pathogen control. Studies have shown the efficacy of certain *Lactobacillus* strains in suppressing or excluding *Salmonella* spp., *Escherichia coli*, *Enterococcus* spp., *Clostridium perfringens* and *Campylobacter* spp.[34, 35].

Here we present a study aimed at manipulating gut microbiota of chickens at-hatch, by providing an inoculum of carefully selected beneficial strains, isolated as able to persistently colonise poultry. At hatch, the initial inoculum shapes the gut microbiota of chickens for life. The first bacteria to enter their intestine, are able to adhere to epithelial cells without competition, rapidly establish, proliferate and set the intestinal environment in terms of metabolite profiles and pH, to best suit their own needs. The first bacterial settlers have the highest influence on the development of intestinal microbiota and subsequent health and productivity of the bird [1, 36]. The development of GIT microbiota in broilers begins immediately post-hatch and is highly variable during early development [36–38]. The diversity and distribution of bacterial species, that make up the GIT microbiota, fluctuates post-hatch and becomes well established as fast as by day 3 [38] or, in a different study, by day 11 [39]. After establishment period, microbiota continues to steadily mature at a slower rate [37]. Not only is the diversity and distribution of the microbiota age dependant, it is also host dependent [40]. Maturity assumes stable microbiota with the ability to resist change, even as severe as antibiotic administration [41], thus we propose that probiotics administered after day 3 will find that the gut is already colonized with near-established community and that only post-hatch administration of probiotics offers the most likely opportunity to achieve permanent colonisation in birds and influence the development of microbiota and thus influence bacterial profile throughout the bird’s life. To test this hypothesis we performed a controlled experiment using culture-free sequencing methodology to observe total bacterial community and inspect the colonisation and success of probiotic’s persistence between at hatch administered probiotic treatment and a sterile PBS inoculated control.

## Materials and methods

### Inoculum preparation

*Lactobacillus* strains were derived from caecal samples collected from birds used in three experiments described by Stanley *et al*. [42]. Briefly, 3 identical broiler trials (each with n = 96) were performed within 5 months in fully environmentally controlled animal facility. Birds were placed in individual metabolic cages from day 15 to day 25 for reliable individual performance indicators. The caecal samples had been stored at -80°C to ensure freeze-thaw resistance of cultured isolates. Samples from healthy birds with the best growth performance were used. The caecal content samples were diluted in MRS broth and plated out onto MRS agar plates. Visually distinct colonies were picked and screened using 16S rRNA gene sequencing. The derived 16S sequences were compared to the NCBI 16S Microbial database to determine the probable genus designation of each isolate and compare isolate to isolate similarity. After this stage the ability to colonise was confirmed *in vivo* and successful colonisers were further identified via Sanger sequencing of near full length 16S amplicons and the species assignment confirmed by sequencing the pheS gene of each isolate. Complete and reliable species identification requires a combination of techniques including DNA hybridisation, sequencing of marker genes, restriction enzyme, sugar utilisation and metabolite profiling, we thus do not claim definite but rather putative species assignment. We aimed to use 3 distinct *Lactobacillus* species, isolated from chicken, which we found abundant and persistent in birds from flocks with extremely different microbiota [36] to investigate whether very early colonising with these chicken-derived *Lactobacillus* strains would provide permanent microbiota modification. The number of bacterial cells of the 3 strains was equalised via pooling of 3 separate inoculums of equal optical density (OD^600^) using freshly plate-grown cells resuspended in PBS.

### At-hatch administration of probiotic mix

Fertilised eggs (Ross 308) were obtained from Bonds hatchery in Toowoomba, Queensland. The eggs were not cleaned or fumigated. The incubator (YBS-FD-440) was cleaned but not fumigated. Hatchlings (n = 11 birds per treatment) were inoculated with either 1ml of sterile PBS or 1ml of inoculum mixture in PBS at 2 hours post-hatch, allowing the birds to dry in the incubator. All birds used in the study hatched within 36 hours. The inoculum mixture contained equal amounts of freshly plate-grown strains provisionally identified as *L*. *ingluviei*, *L*. *agilis* and *L*. *reuteri*. The two groups of birds were then placed in two separate pens on wood shavings in a temperature controlled room. Each pen was 1.2m x 1.2m. The temperature in the room was set as per breed recommendations and 14 hours light, 10 hours dark schedule was used after removing brooding lamps at 5 days of age. Feed used was antibiotic and anticoccidials free chicken starter crumbles (Blue Ribbon Stockfeed, Rockhampton, Queensland). Individual birds were weighed daily, and pen feed intake also measured daily, for 28 days. Excreta samples were taken for microbiota analysis at 14 days, and before slaughtering of the birds at 28 days of age. The samples were collected by placing wide wire confining divider into the pen, separating one bird from others without handling or removing the bird from the pen and waiting for the bird to pass the fresh excreta. Care was taken that fresh excreta taken as a sample was free of any caecal matter by visual observation. In addition, caecal contents and ileal mucosa scrapings were collected at 28 days and prepared for microbiota analysis.

DNA was extracted using Bioline ISOLATE Faecal DNA Kit (#BIO-52038) according to the manufacturer’s instructions and 16S variable regions V3-V4 were amplified. DNA was amplified using Q5 DNA polymerase (New England Biolabs). Sequencing was performed on an Illumina MiSeq system (2 x 300 bp) using the dual-indexing, variable spacer, method detailed by Fadrosh *et al*. [43]. The quality filtered sequences were analysed in QIIME 1.9.1 software [44] using QIIME default parameters unless stated otherwise. OTUs were picked using UCLUST algorithm (Edgar 2010) at 97% sequence identity and inspected for chimeric sequences using Pintail [45] and taxonomy assigned with GreenGenes database [46]. Additional taxonomic assignments were done using blastn against the NCBI 16S database. Data were analysed and visualised using Calypso [47]. The OTU table was filtered to remove low abundance OTUs (less than 0.01%), square root transformed and TSS normalised. Significance of microbiota differences between probiotic and control was calculated using Wilcoxon test via biomarker discovery function in Calypso [47]. Annotated sequencing dataset used in this study is publically available on the MG-RAST database under the project number mgp83960 and the library accession number mgl639405.

### Ethics statement

This study was approved by the Animal Ethics Committee of Central Queensland University (A1409-318). The *Lactobacillus* isolation experiment, including *in vivo* testing of colonisation success declared in this manuscript, was approved by the Animal Ethics Committees of RMIT University (1508). All animal work was conducted in accordance with “The Australian code for the use of animals for scientific purposes” document.

## Results

### Colonisation success

**Fig 1:**
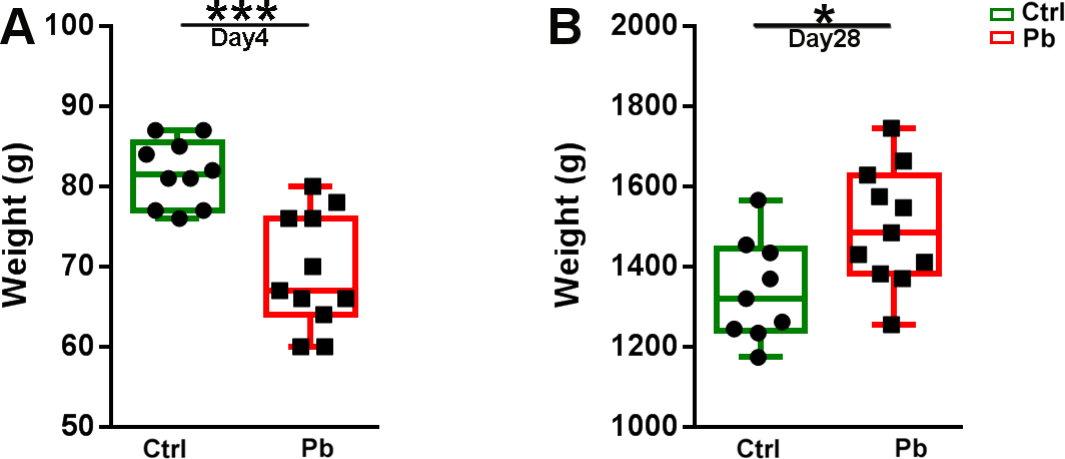
**Weights of the birds at 4 (A) and 28 days (B).** Control PBS inoculated birds were significantly heavier between the days 2 and 6 post hatch. Between day 6 and 24 there were no significant differences, however, probiotic inoculated group became significantly heavier starting from day 24 to the end of the trail.

There were significant differences in weights of birds between probiotic treated and untreated birds. The control birds grew faster at the start of the trial and were heavier during the first week, significantly so between days 2 and 6 (Fig 1A). However, after day 6 there were no significant differences between the treatments until day 24, when the probiotic treated birds exceeded weights of the controls, remaining significantly heavier at 28 days of age, at the time of the trial termination (Fig 1B).

**Fig 2:**
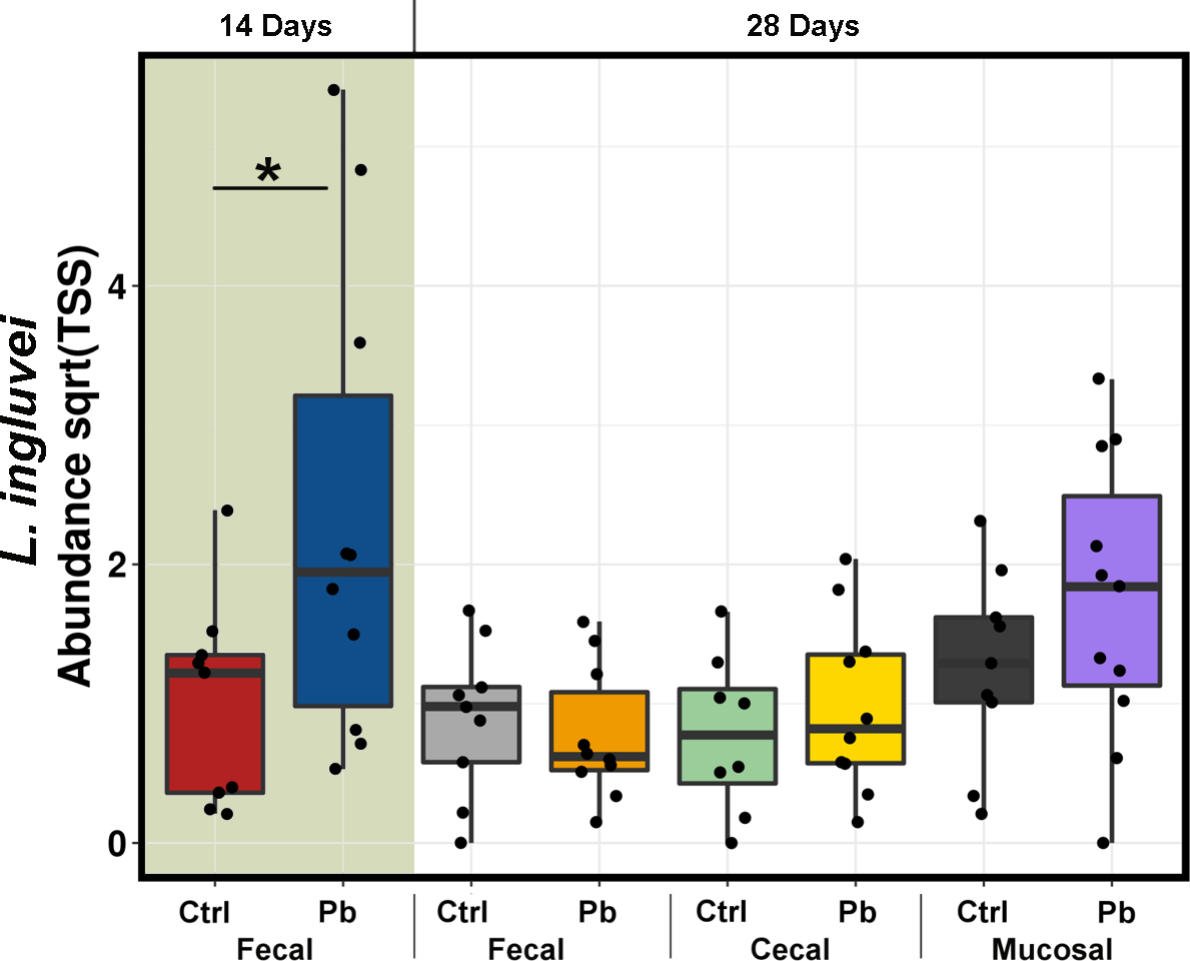
TSS normalised, square root transformed abundance of inoculated *L*. *ingluviei*. Out of the 3 OTUs detected in inoculum, only one, identical to *L*. *ingluviei* was detected in birds using sequencing methodology and was present in all birds across all sampling points. Legend: Pb = probiotic, Ctrl = PBS control.

OTUs corresponding to the three inoculated strains were identified in the sequenced inoculum samples, however, only one of these OTUs, identical to *L*. *ingluvei* strain, was able to persist until the end of the experiment while the other two were not detected at any stage of sampling, including ileal mucosa and caecal samples (Fig 2). There was a contact of inoculum strains to control birds due to the shared shed environment, despite the wire pens providing physical separation. This allowed comparing at-hatch large dose inoculation to environmental colonisation between the two groups. There were significant differences in the abundance of *L*. *ingluvei* OTU across the treatments, however, the only meaningful significant difference, comparing the control and probiotic at the same point of sampling, was at 14 days of age when the inoculated strain was still at a higher level (P = 0.043) than in control in collected excreta samples. By 28 days, when we additionally sampled caecal content and ileal mucosa, the significance between the treatments diminished and inoculated *L*. *ingluvei* OTU was noticeably higher only in probiotic treated illeal mucosa, however, not significantly (P = 0.33). There were no significant differences in Richness or Evenness index between the probiotic and control groups although there were differences between the excreta, caecal and mucosal samples as anticipated.

**Fig 3:**
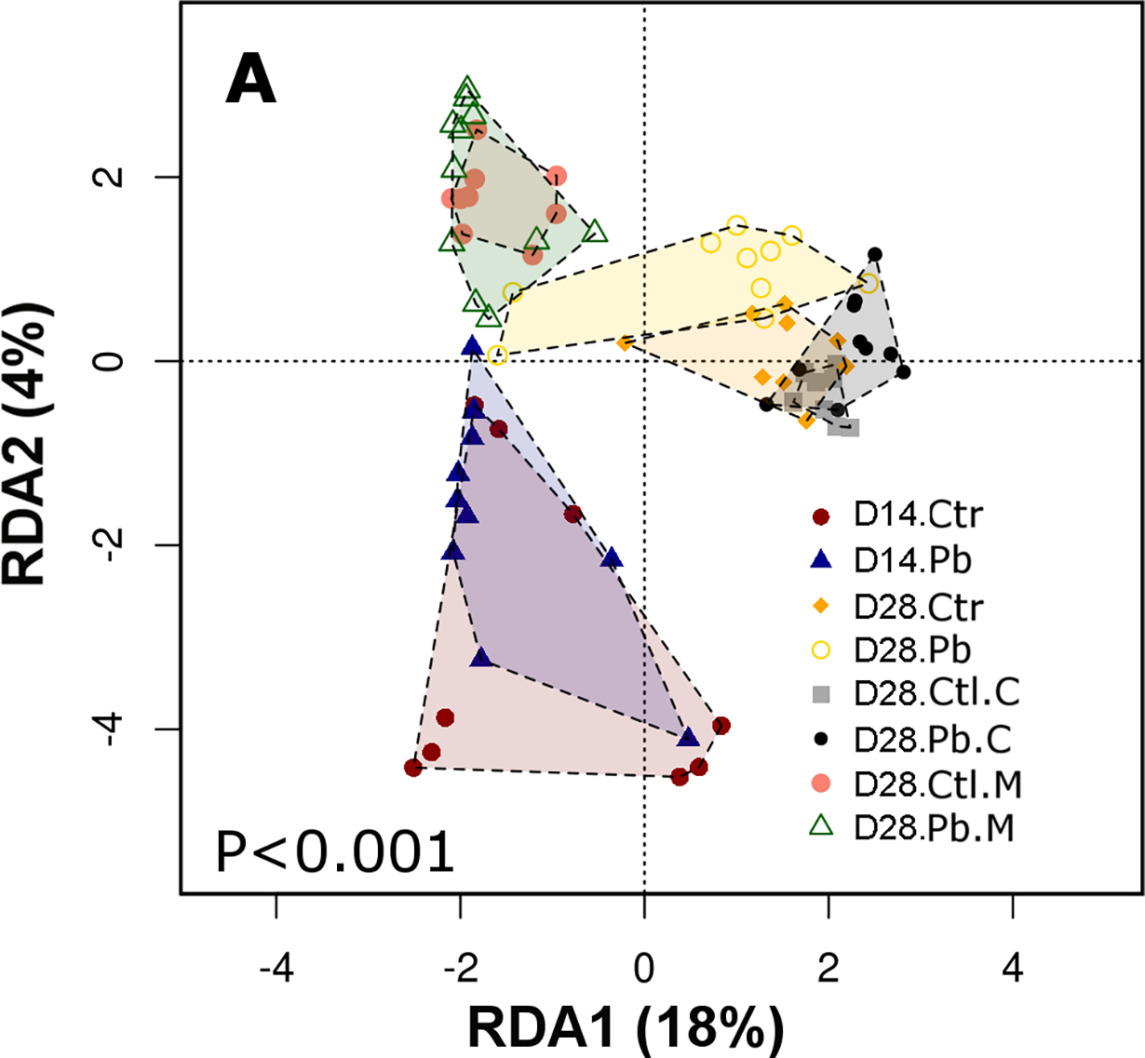
Redundancy analysis (RDA) plot showing group to group microbial community differences between the treatments, timepoints and sampling origins. Legend: D14 = 14 days, D28 = 28 days, Pb = probiotic, Ctr = PBS control, C = caecal, M = ileal mucosa.

Multivariate analysis additionally showed significant differences in microbiota structure between the groups and by both redundancy analysis (RDA, Fig 3) and Adonis statistics on Weighted Unifrac, P<0.001. We further used Weighted Unifrac and Adonis multivariate statistics to identify variables influencing the microbiota differences. Based on Adonis, bird sex had no significance (P = 0.825), while probiotic administration significantly affected microbiota (P = 0.037). The different microbiota origins (caecal and ileal mucosa or excreta samples) likewise had significantly different communities (P = 0.015). This analysis took into account all sample origins and time points (all 76 sequenced samples as presented in Fig 2). However, separate analysis was performed for each individual time-point/sample origin matched probiotic and control samples (for example excreta control vs excreta probiotic, both 14 days, n = 10 each) where no significant influence of probiotic was detected on individual weekly comparisons with smaller sample size.

### Changes in excreta microbiota at 14 and 28 days post inoculum

**Fig 4:**
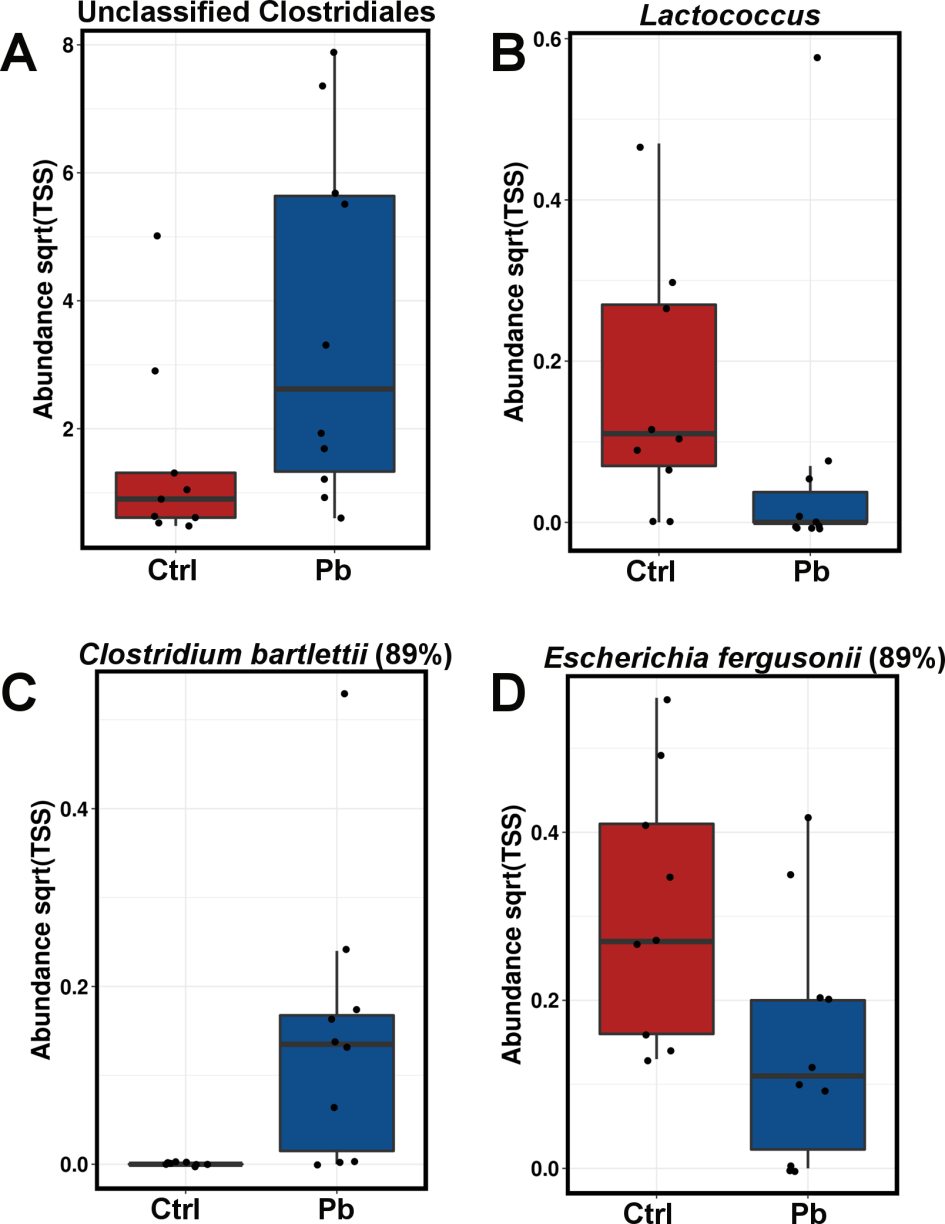
Phylotypes significantly altered by probiotic administration 14 days post hatch. Complete data with significance values is provided in Tables A and B in S1 File. Blastn best hits against 16S Microbial database and % ID are given as a guide.

**Fig 5:**
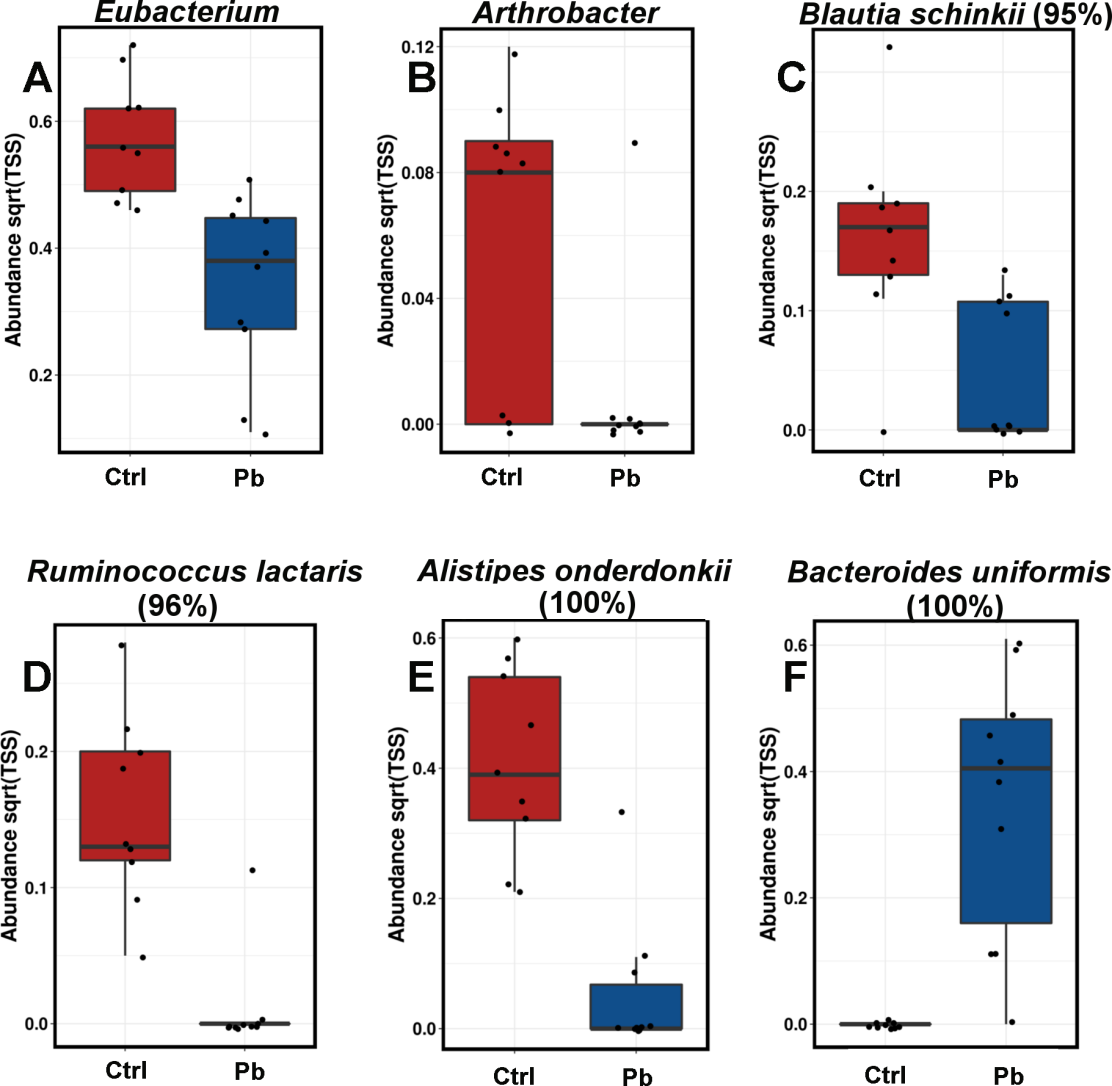
Boxplots showing some of the excreta phylotypes altered in abundance by at-hatch probiotic administration 28 days post hatch. Complete data with significance values is provided in Tables C and D in S1 File. Blastn best hits against 16S Microbial database and % ID are given as a guide.

Two weeks after the oral inoculation overall microbiota did not differ significantly between control and inoculated group (Adonis on Weighted Unirac P = 0.203, n = 10), however significant changes were detected (Wilcoxon P<0.05) in individual taxa. At the genus level (Table A in S1 File), the abundance of unclassified Planococcaceae and Clostridiales (Fig 4A) was increased in the probiotic inoculated birds, while genera *Pseudoclavibacter*, *Nocardiopsis*, *Burkholderia* and *Brevundimonas* were detected only in faeces of probiotic inoculated birds. *Lactococcus* (Fig 4B) was significantly reduced in the probiotic treated birds. Among the OTUs there were a number of significantly altered taxa, mostly OTUs belonging to species of the significantly affected genera listed above with a high number of clostridia-related OTUs (Fig 5C) increased in probiotic treated group. Clostridia related species that were most similar to the clostridial OTUs increased by probiotic were not related to pathogenic “true” Clostridiaceae clostridia, but aligned with more recently isolated species, some of which are considered beneficial via high short chain acid (SCFA) production. Additionally Enterobacteriaceae OTU, most similar to *Escherichia fergusonii* (Fig 4D) and *Shigella sonnei* (89%) was reduced in abundance in probiotic treated group. The list of genera and OTUs altered at 14 days after at hatch administration of probiotic with significance levels is given in Tables A and B in S1 File.

Two weeks later, at 28 days of bird’s age, the excreta microbiota was different from the one at 14 days. This is expected due to microbiota maturation and development changes [37]. The level of changes according to Weighted Unifrac and ADONIS statistics was comparable in control (control, 14 vs 28 days; P = 0.002) and in probiotic treated birds (probiotic, 14 vs 28 days; P = 0.002). The differences between the probiotic and control microbial communities at 28 days of age were fewer (Adonis P = 0.841) in excreta samples with only *Eubacterium* and *Arthrobacter* genera different between the treatments (Fig 5A and 5B, Table C in S1 File) and OTUs with %ID closest to *Blautia*, *Ruminococcus*, *Alistipes* and *Bacteroides* species (Fig 5C–5F, Table D in S1 File).

**Fig 6:**
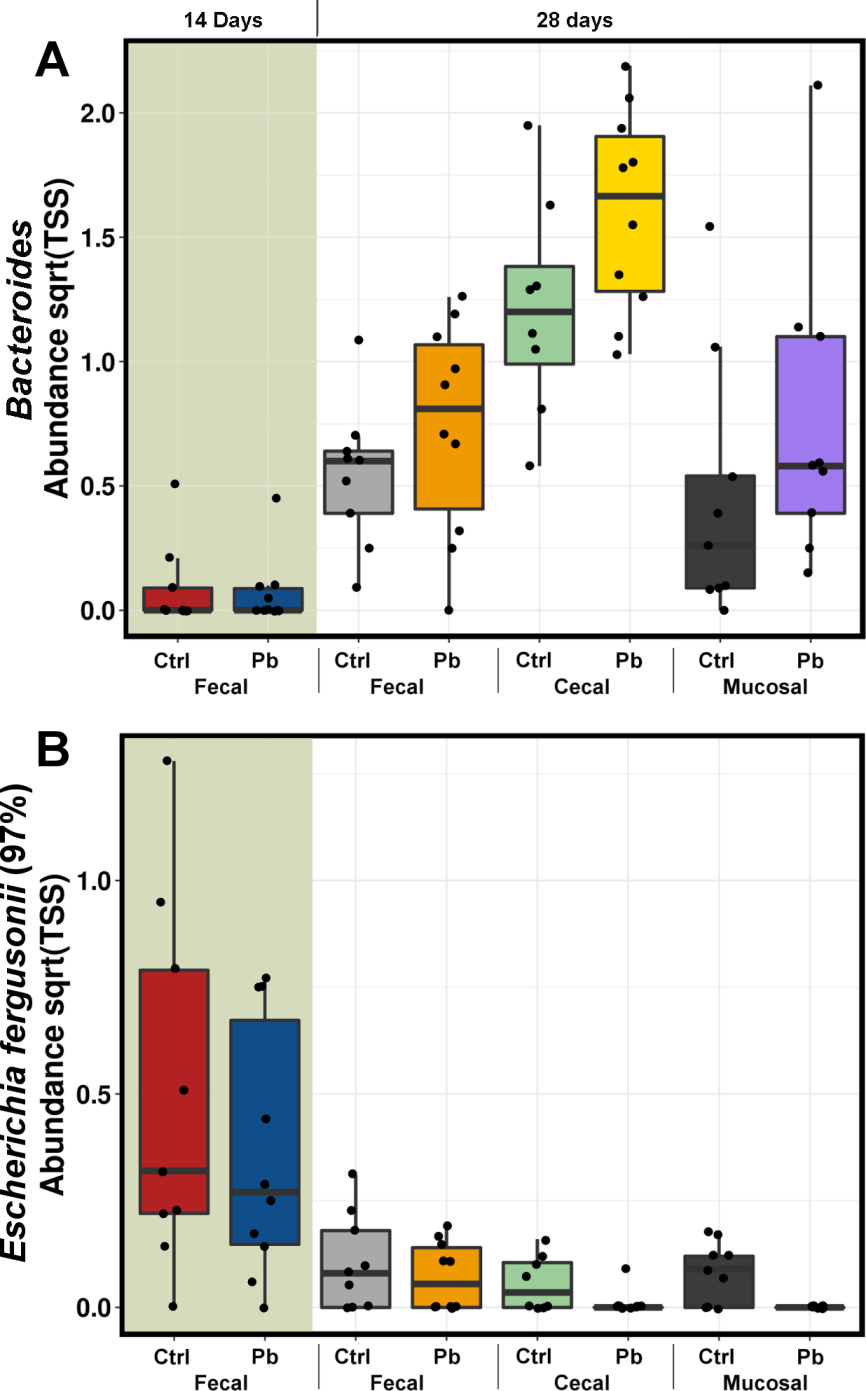
TSS normalised, square root transformed abundance of genus *Bacteroides* and an OTU most similar (blastn on 16S Microbial database) to *Escherichia fergusonii*.

In addition to these two genera also significant in caeca, the caecal community additionally differed significantly in *Brachybacterium*, *Coprobacillus* and *Alistipes* (Figure A in S1 File, Tables E and F in S1 File) and ileal mucosa had no overlap with excreta or caecal differences with increased *Weissella* and *Paracoccus* and reduced unclassified Clostridiales in probiotic treated birds (Figure A in S1 File, Tables G and H in S1 File). Both caecal (P = 0.461) and ileum (P = 0.445) mucosal microbiotas were not significantly altered between treatments at 28 days. However at the OTU level there were some consistent changes noticed. A number of *Bacteroides uniformis* assigned OTUs were significantly increased in probiotic treated birds across the sampling origins contributing to *Bacteroides* genus, being highly significantly differential between the groups (ANOVA P = 3E^-12^) (Fig 6A). Likewise, an OTU, most similar to *Escherichia fergusonii* (97%), the most significantly differential OTU in ileum, showed consistency across the time-points and sampling origins (ANOVA P = 5.8E^-7^, Fig 6B) as reduced in probiotic treated birds.

## Discussion

Probiotic strains are highly host specific and often strains isolated from one animal species cannot colonise other closely related animals [48]. However, strains isolated for human use were often marketed for agricultural animals until recent advances in total microbiota profiling and sequencing technology revealed a wealth of knowledge on microbiota interactions with the host. Although OTU identical to only one of the 3 inoculated strains 16S amplicon sequence was detected in experimental birds, it needs to be noted that 16S sequencing based microbiota profiling studies can detect OTUs present in excreta material higher than 10^6^ cell per g [49] and that based on changes observed in total microbiota profiles between the control and probiotic administered group, it is also likely that the inoculated species remained to some extent in the community, and that deeper sequencing could have determined the degree of success in colonisation. Additionally, it was reported that some strains can help others colonise without establishing strong presence themselves and also that multi-strain probiotics are generally more efficient than single strains they are comprised of [50] although strains could not colonise with the same success.

Our data show that there are clear differences between probiotic-inoculated and control birds with effects often reproducible even between physiologically different gut sampling origins (Fig 6). While the presence of the best colonising inoculated strain was higher in earlier days reducing by day 28, the probiotic inoculation had lasting effects on the development of the community rather than establishing dominance.

Comparable phylotypes were altered in the probiotic inoculated group in both excreta and caecal samples as expected [51]. These changes in the probiotic group consisted of reduction in *Alistipes* and *Ruminococcus* related species. Not much is known about the roles of *Alistipes*, while *Ruminococcus* are generally considered as beneficial bacteria. However, most of the *Ruminococcus*–annotated OTUs were also highly similar with *Clostridium* species from *Ruminococcaceae* family, these are often difficult to resolve with 16S based taxonomy and may require additional identification. Probiotic treatment also increased *Bacteroides uniformis* species, (some OTUs were 100% identical in sequence across the amplified region), in both excreta and caecal samples. Some of these OTUs were completely absent from control faeces and caeca. *Bacteroides uniformis* is known to have the potential to degrade the isoflavones in the gut [52], and significantly improve metabolic and immunological dysfunction in mice with diet induced obesity [53]. However, some antibiotic resistant strains may have pathogenic potential [54]. There was also an indication that at-hatch inoculation of *L*. *ingluvei* was able to reduce *Shigella / Escherichia* related OTUs. These two genera cannot be resolved using partial 16S amplicons. It was previously reported that *Lactobacillus acidophilus* was able to inhibit growth of *Escherichia coli* in chickens [55]. However, in our data, this inhibition was not detected during classic ongoing probiotic administration but instead, it was confirmed 28 days post single probiotic dose inoculation. Total removal or depletion of *Escherichia* or other enterobacteria from intestinal mucosa may have clinical significance.

Another promising outcome was an increase of *Weissella* species in mucosal bacterial communities. We have previously reported the depletion of *Weissella confusa* is associated with necrotic enteritis in chicken [56]. *Weissella confusa* is a heterofermentative lactic acid bacteria, previously classified as *Lactobacillus* [57], used in traditional fermentations [58], and is also used as probiotic [59, 60]. More *in vitro* and *in vivo* investigations are needed to validate the ability of these strains to stimulate *Weissella* and other mucosa protective species as probiotics are often associated with repair of gut mucosal disruptions such as diarrhoea [61].

## Conclusions

The results indicate that early inoculation of probiotic strains can influence intestinal microbiota and has the potential to improve weight gain via microbiota modifications. Our data confirm the clear difference between at-hatch administration of beneficial strain compared to the natural acquisition of the same strain from the environment. The way of inoculum preparation (fresh, exponentially growing vs freeze-dried cells for example), chicken breed and shed/farm resident microbiota are all expected to play a role in the outcome of probiotic treatment and should be further investigated.

## Supporting information

S1 File This file contains Figure A and Tables A-H.
(PDF)

## References

[1] Stanley D, Hughes R, Moore R. Microbiota of the chicken gastrointestinal tract: influence on health, productivity and disease. Applied Microbiology and Biotechnology. 2014;98(10):4301–10. 10.1007/s00253-014-5646-2 24643736

[2] Stanley D, Keyburn AL, Denman SE, Moore RJ. Changes in the caecal microflora of chickens following Clostridium perfringens challenge to induce necrotic enteritis. Veterinary Microbiology. 2012;159(1–2):155–62. 10.1016/j.vetmic.2012.03.032. 22487456

[3] Bailey R. Gut Health in Poultry—The World Within. Aviagen, editor. Huntsville USA: Aviagen; 2013.

[4] Stanley D, Denman SE, Hughes RJ, Geier MS, Crowley TM, Chen H. Intestinal microbiota associated with differential feed conversion efficiency in chickens. Appl Microbiol Biotechnol. 2012;96 10.1007/s00253-011-3847-5. 22249719

[5] Mohan B, Kadirvel R, Natarajan A, Bhaskaran M. Effect of probiotic supplementation on growth, nitrogen utilisation and serum cholesterol in broilers. British poultry science. 1996;37(2):395–401. 10.1080/00071669608417870 8773848

[6] Jin LZ, Ho YW, Abdullah N, Jalaludin S. Growth performance, intestinal microbial populations, and serum cholesterol of broilers fed diets containing Lactobacillus cultures. Poultry science. 1998;77(9):1259–65. 10.1093/ps/77.9.1259 9733111

[7] Alkhalf A, Alhaj M, Al-homidan I. Influence of probiotic supplementation on blood parameters and growth performance in broiler chickens. Saudi Journal of Biological Sciences. 2010;17(3):219–25. 10.1016/j.sjbs.2010.04.005 PMC3730717. 23961081 PMC3730717

[8] Haghighi HR, Gong J, Gyles CL, Hayes MA, Zhou H, Sanei B, et al Probiotics Stimulate Production of Natural Antibodies in Chickens. Clinical and Vaccine Immunology. 2006;13(9):975–80. 10.1128/CVI.00161-06 PMC1563569. 16960107 PMC1563569

[9] Guinane C, Cotter PD. Role of the gut microbiota in health and chronic gastrointestinal disease: understanding a hidden metabolic organ. Therapeutic Advances in Gastroenterology. 2013;6(4):295–308. 10.1177/1756283X13482996 PMC3667473. 23814609 PMC3667473

[10] Ohland C, Jobin C. Microbial Activities and Intestinal Homeostasis: A Delicate Balance Between Health and Disease. Cellular and Molecular Gastroenterology and Hepatology. 2014;1(1):28–40. 10.1016/j.jcmgh.2014.11.004 25729763 PMC4339954

[11] Lan Y, Verstegen MWA, Tamminga S, Williams BA. The role of the commensal gut microbial community in broiler chickens. World's Poultry Science Journal. 2005;61(01):95–104. 10.1079/WPS200445

[12] Ridaura VK, Faith JJ, Rey FE, Cheng J, Duncan AE, Kau AL, et al Cultured gut microbiota from twins discordant for obesity modulate adiposity and metabolic phenotypes in mice. Science (New York, NY). 2013;341(6150): 10.1126/science.1241214 PMC3829625. 24009397 PMC3829625

[13] Rinttilä T, Apajalahti J. Intestinal microbiota and metabolites—Implications for broiler chicken health and performance1. The Journal of Applied Poultry Research. 2013;22(3):647–58. 10.3382/japr.2013-00742

[14] Walker WA. Mechanisms of Action of Probiotics. Clinical Infectious Diseases. 2008;46:S87–S91. 10.1086/523335 . 18181730

[15] Kaushik JK, Kumar A, Duary RK, Mohanty AK, Grover S, Batish VK. Functional and Probiotic Attributes of an Indigenous Isolate of Lactobacillus plantarum. PLoS ONE. 2009;4(12):e8099 10.1371/journal.pone.0008099 PMC2779496. 19956615 PMC2779496

[16] Cotter PD, Ross RP, Hill C. Bacteriocins [mdash] a viable alternative to antibiotics? Nat Rev Micro. 2013;11(2):95–105. 10.1038/nrmicro2937 23268227

[17] Aliakbarpour H, Chamani M, Rahimi G, Sadeghi A, Qujeq D. The Bacillus subtilis and Lactic Acid Bacteria Probiotics Influences Intestinal Mucin Gene Expression, Histomorphology and Growth Performance in Broilers. Asian-Australasian Journal of Animal Sciences. 2012;25(9):1285–93. 10.5713/ajas.2012.12110 PMC4092943. 25049692 PMC4092943

[18] La Ragione RM, Woodward MJ. Competitive exclusion by Bacillus subtilis spores of Salmonella enterica serotype Enteritidis and Clostridium perfringens in young chickens. Veterinary Microbiology. 2003;94(3):245–56. 10.1016/S0378-1135(03)00077-4. 12814892

[19] Murry AC, Murry AC Jr, Hinton A, Morrison H. Inhibition of Growth of Escherichia coli, Salmonella typhimurium, and Clostridia perfringens on Chicken Feed Media by Lactobacillus salivarius and Lactobacillus plantarum. International journal of poultry science. 2004;3(9):603–7.

[20] Adlerberth MC, Poilane Isabelle, Wold Agnes, Collignon Anne, Ingegerd. Mechanisms of colonisation and colonisation resistance of the digestive tract part 1: bacteria/host interactions. Microbial ecology in health and disease. 2000;12(2):223–39.

[21] Mohan V. The role of probiotics in the inhibition of Campylobacter jejuni colonization and virulence attenuation. European Journal of Clinical Microbiology & Infectious Diseases. 2015;34(8):1503–13. 10.1007/s10096-015-2392-z 25934376

[22] Fuller R. Probiotics in man and animals. J Appl Bacteriol. 1989;66(5):365–78. Epub 1989/05/01. . 2666378

[23] Sanders ME. Probiotics: considerations for human health. Nutr Rev. 2003;61(3):91–9. . 12723641 10.1301/nr.2003.marr.91-99 PMC7167708

[24] Conway P. Function and regulation of the gastrointestinal microbiota of the pig. PUBLICATION-EUROPEAN ASSOCIATION FOR ANIMAL PRODUCTION. 1994;80:231–.

[25] Dunne C, Murphy L, Flynn S, O’Mahony L, O’Halloran S, Feeney M, et al Probiotics: from myth to reality. Demonstration of functionality in animal models of disease and in human clinical trials Lactic Acid Bacteria: Genetics, Metabolism and Applications: Springer; 1999 p. 279–92. 10532384

[26] Kailasapathy K, Chin J. Survival and therapeutic potential of probiotic organisms with reference to Lactobacillus acidophilus and Bifidobacterium spp. Immunology and cell biology. 2000;78(1):80–8. 10.1046/j.1440-1711.2000.00886.x 10651933

[27] Pan D, Yu Z. Intestinal microbiome of poultry and its interaction with host and diet. Gut Microbes. 2014;5(1):108–19. 10.4161/gmic.26945 PMC4049927. 24256702 PMC4049927

[28] Bbosa GS, Mwebaza N, Odda J, Kyegombe DB, Ntale M. Antibiotics/antibacterial drug use, their marketing and promotion during the post-antibiotic golden age and their role in emergence of bacterial resistance. Health. 2014;Vol.06No.05:16 10.4236/health.2014.65059

[29] M'Sadeq Shawkat, Wu Shubiao, Swick Robert, Choct M. Towards the control of necrotic enteritis in broiler chickens with in-feed antibiotics phasing-out worldwide. Animal Nutrition. 2015;1(1):1–11. 10.1016/j.aninu.2015.02.004. 29766984 PMC5884463

[30] Steer T, Carpenter H, Tuohy K, Gibson GR. Perspectives on the role of the human gut microbiota and its modulation by pro-and prebiotics. Nutrition research reviews. 2000;13(02):229–54. 19087441 10.1079/095442200108729089

[31] Fooks L, Gibson G. Probiotics as modulators of the gut flora. British Journal of Nutrition. 2002;88(S1):s39–s49. 12215180 10.1079/BJN2002628

[32] Schiffrin E, Blum S. Interactions between the microbiota and the intestinal mucosa. European journal of clinical nutrition. 2002;56:S60–4. 10.1038/sj.ejcn.1601489 12142966

[33] Kosin B, Rakshit SK. Microbial and processing criteria for production of probiotics: a review. Food Technology and Biotechnology. 2006;44(3):371–9.

[34] Gusils C, Oppezzo O, Pizarro R, Gonzalez S. Adhesion of probiotic lactobacilli to chick intestinal mucus. Canadian journal of microbiology. 2003;49(7):472–8. 10.1139/w03-055 14569288

[35] Lee Y-J, Yu W-K, Heo T-R. Identification and screening for antimicrobial activity against Clostridium difficile of Bifidobacterium and Lactobacillus species isolated from healthy infant faeces. International journal of antimicrobial agents. 2003;21(4):340–6. 12672580 10.1016/s0924-8579(02)00389-8

[36] Stanley D, Geier MS, Hughes RJ, Denman SE, Moore RJ. Highly variable microbiota development in the chicken gastrointestinal tract. PLoS One. 2013;8(12):e84290 10.1371/journal.pone.0084290 24391931 PMC3877270

[37] Donaldson EE, Stanley D, Hughes RJ, Moore RJ. The time-course of broiler intestinal microbiota development after administration of cecal contents to incubating eggs. PeerJ. 2017;5:e3587 10.7717/peerj.3587 28740754 PMC5522604

[38] Apajalahti J, Kettunen A, Graham H. Characteristics of the gastrointestinal microbial communities, with special reference to the chicken. World's Poultry Science Journal. 2004;60:223–32.

[39] van der Wielen PW, Keuzenkamp DA, Lipman LJ, van Knapen F, Biesterveld S. Spatial and temporal variation of the intestinal bacterial community in commercially raised broiler chickens during growth. Microb Ecol. 2002;44(3):286–93. Epub 2002/09/10. 10.1007/s00248-002-2015-y . 12219265

[40] Fujisaka S, Ussar S, Clish C, Devkota S, Dreyfuss JM, Sakaguchi M, et al Antibiotic effects on gut microbiota and metabolism are host dependent. The Journal of Clinical Investigation. 2016;126(12):4430–43. 10.1172/JCI86674 PMC5127688. 27775551 PMC5127688

[41] Catherine AL, Jesse IS, Jeffrey IG, Janet KJ, Rob K. Diversity, stability and resilience of the human gut microbiota. Nature. 2012;489(7415):220 10.1038/nature11550 22972295 PMC3577372

[42] Stanley D, Hughes RJ, Geier MS, Moore RJ. Bacteria within the gastrointestinal tract microbiota correlated with Improved growth and feed conversion: challenges presented for the identification of performance enhancing probiotic bacteria. Frontiers in microbiology. 2016;7(187). 10.3389/fmicb.2016.00187 26925052 PMC4760072

[43] Fadrosh DW, Ma B, Gajer P, Sengamalay N, Ott S, Brotman RM, et al An improved dual-indexing approach for multiplexed 16S rRNA gene sequencing on the Illumina MiSeq platform. Microbiome. 2014;2(1):6 10.1186/2049-2618-2-6 24558975 PMC3940169

[44] Caporaso JG, Kuczynski J, Stombaugh J, Bittinger K, Bushman FD, Costello EK, et al QIIME allows analysis of high-throughput community sequencing data. Nature methods. 2010;7(5):335–6. 10.1038/nmeth.f.303 20383131 PMC3156573

[45] Ashelford KE, Chuzhanova NA, Fry JC, Jones AJ, Weightman AJ. At least 1 in 20 16S rRNA sequence records currently held in public repositories is estimated to contain substantial anomalies. Appl Environ Microbiol. 2005;71(12):7724–36. Epub 2005/12/08. 71/12/7724 [pii] 10.1128/AEM.71.12.7724-7736.2005 16332745 PMC1317345

[46] DeSantis TZ, Hugenholtz P, Larsen N, Rojas M, Brodie EL, Keller K, et al Greengenes, a chimera-checked 16S rRNA gene database and workbench compatible with ARB. Appl Environ Microbiol. 2006;72(7):5069–72. Epub 2006/07/06. 72/7/5069 [pii] 10.1128/AEM.03006-05 16820507 PMC1489311

[47] Zakrzewski M, Proietti C, Ellis JJ, Hasan S, Brion MJ, Berger B, et al Calypso: a user-friendly web-server for mining and visualizing microbiome-environment interactions. Bioinformatics. 2016 10.1093/bioinformatics/btw725 . 28025202 PMC5408814

[48] Tannock GW, Miller JR, Savage DC. Host specificity of filamentous, segmented microorganisms adherent to the small bowel epithelium in mice and rats. Appl Environ Microbiol. 1984;47(2):441–2. 6712214 10.1128/aem.47.2.441-442.1984 PMC239693

[49] Hiergeist A, Glasner J, Reischl U, Gessner A. Analyses of Intestinal Microbiota: Culture versus Sequencing. ILAR J. 2015;56(2):228–40. 10.1093/ilar/ilv017 . 26323632

[50] Chapman CM, Gibson GR, Rowland I. Health benefits of probiotics: are mixtures more effective than single strains? Eur J Nutr. 2011;50(1):1–17. 10.1007/s00394-010-0166-z . 21229254

[51] Stanley D, Geier MS, Chen H, Hughes RJ, Moore RJ. Comparison of fecal and cecal microbiotas reveals qualitative similarities but quantitative differences. BMC Microbiol. 2015;15(1):51 10.1186/s12866-015-0388-6 25887695 PMC4403768

[52] Renouf M, Hendrich S. Bacteroides uniformis is a putative bacterial species associated with the degradation of the isoflavone genistein in human feces. J Nutr. 2011;141(6):1120–6. 10.3945/jn.111.140988 . 21525249

[53] Gauffin Cano P, Santacruz A, Moya A, Sanz Y. Bacteroides uniformis CECT 7771 ameliorates metabolic and immunological dysfunction in mice with high-fat-diet induced obesity. PLoS One. 2012;7(7):e41079 10.1371/journal.pone.0041079 22844426 PMC3406031

[54] Zar FA, Bond EJ. Infection with clindamycin-resistant bacteroides uniformis. Chemotherapy. 1985;31(1):29–33. 10.1159/000238310 . 3971777

[55] Watkins BA, Miller BF, Neil DH. In vivo inhibitory effects of Lactobacillus acidophilus against pathogenic Escherichia coli in gnotobiotic chicks. Poult Sci. 1982;61(7):1298–308. 10.3382/ps.0611298 . 6813835

[56] Stanley D, Keyburn AL, Denman SE, Moore RJ. Changes in the caecal microflora of chickens following Clostridium perfringens challenge to induce necrotic enteritis. Veterinary Microbiology. 2012;159:155–62. 10.1016/j.vetmic.2012.03.032 22487456

[57] Bjorkroth KJ, Schillinger U, Geisen R, Weiss N, Hoste B, Holzapfel WH, et al Taxonomic study of Weissella confusa and description of Weissella cibaria sp. nov., detected in food and clinical samples. Int J Syst Evol Microbiol. 2002;52(Pt 1):141–8. Epub 2002/02/12. 10.1099/00207713-52-1-141 . 11837296

[58] Kim M, Chun J. Bacterial community structure in kimchi, a Korean fermented vegetable food, as revealed by 16S rRNA gene analysis. Int J Food Microbiol. 2005;103(1):91–6. Epub 2005/08/09. S0168-1605(05)00076-0 [pii] 10.1016/j.ijfoodmicro.2004.11.030 . 16084269

[59] Nam H, Ha M, Bae O, Lee Y. Effect of Weissella confusa strain PL9001 on the adherence and growth of Helicobacter pylori. Appl Environ Microbiol. 2002;68(9):4642–5. Epub 2002/08/30. 10.1128/AEM.68.9.4642-4645.2002 12200324 PMC124069

[60] Moreira JL, Mota RM, Horta MF, Teixeira SM, Neumann E, Nicoli JR, et al Identification to the species level of Lactobacillus isolated in probiotic prospecting studies of human, animal or food origin by 16S-23S rRNA restriction profiling. BMC Microbiol. 2005;5:15 Epub 2005/03/25. 1471-2180-5-15 [pii] 10.1186/1471-2180-5-15 15788104 PMC1079852

[61] Alam A, Leoni G, Quiros M, Wu H, Nusrat A, Neish A. O-012 The Intestinal Wound Regeneration Modulates Mucosal Microenvironment to Stimulate Expansion of a Local Pro-restitutive Microbiota. Inflamm Bowel Dis. 2016;22 Suppl 1:S4 10.1097/01.MIB.0000480098.23732.ba .

