# Supplementary material for: At-hatch administration of probiotic to chickens can introduce beneficial changes in gut microbiota
Source: PLoS One. 2018 Mar 23;13(3):e0194825. doi: 10.1371/journal.pone.0194825 (PMC5865720; doi:10.1371/journal.pone.0194825)
Supplement: S1 File — This file contains Figure A and Tables A-H. (PDF) [file pone.0194825.s001.pdf]

At-hatch administration of probiotic to chickens can introduce permanent and beneficial changes in gut microbiota

Running Title: Early administration of probiotics

Stephen Baldwin[1], Robert J. Hughes[3,4], Thi Hao Van[2], Robert J. Moore[2] and Dragana Stanley[2§]

[1]Central Queensland University, Institute for Future Farming Systems, Rockhampton, Queensland, Australia.

[2]RMIT University, School of Science, Bundoora, Victoria, Australia.

[3]South Australian Research and Development Institute, Roseworthy, South Australia, Australia.

[4]The University of Adelaide, School of Animal and Veterinary Sciences Roseworthy, South Australia, Australia.

[§]Correspondence:

Dragana Stanley

Central Queensland University

Institute for Future Farming Systems

630 Ibis Avenue, CQIRP, North Rockhampton, QLD, Australia

Email: D.Stanley@cqu.edu.au

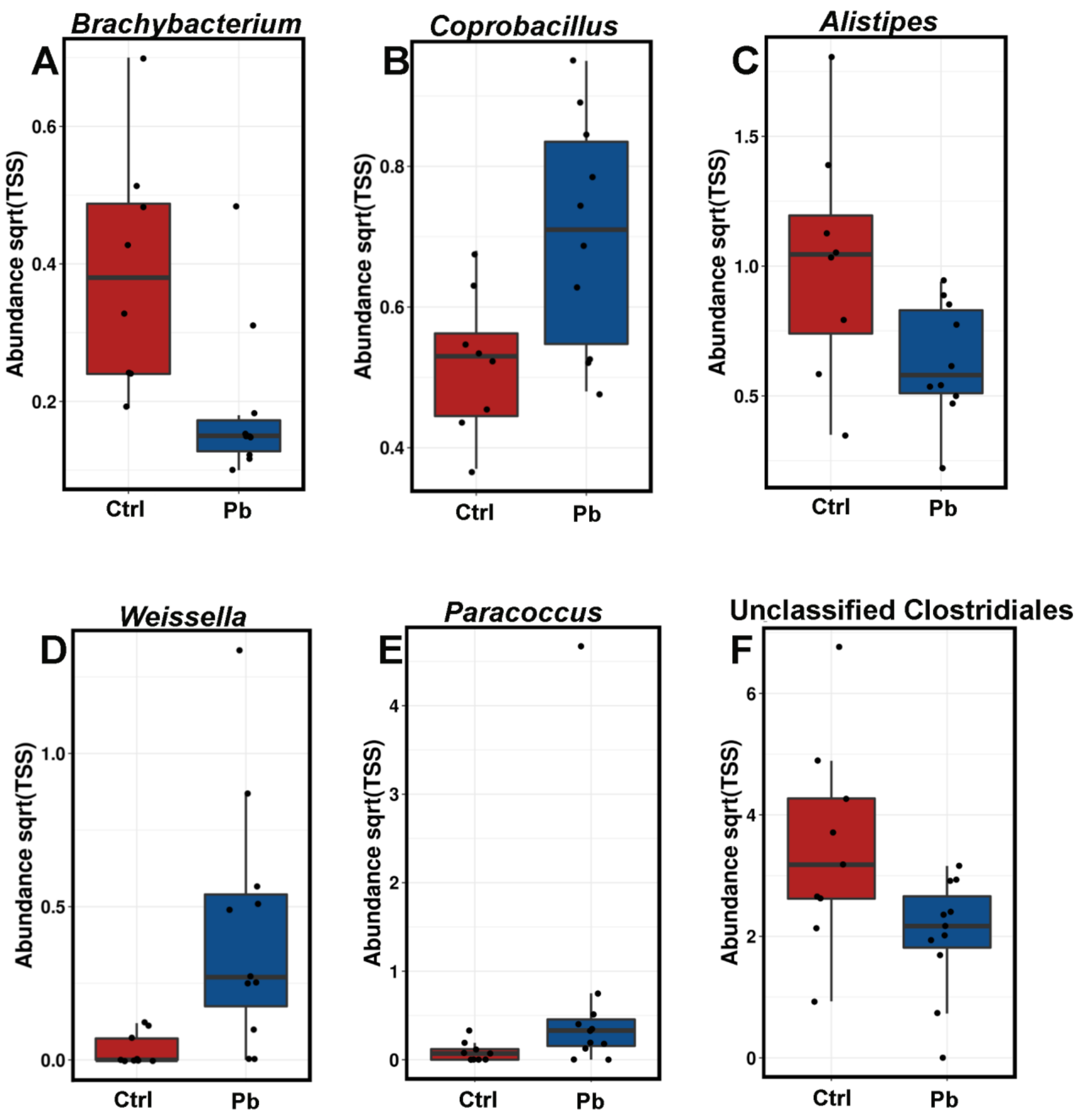

Figure A: Genera significantly different between probiotic inoculated birds and control in caecal content (A-C) and in ileal mucosa (D-E) microbial communities.

The full list of affected genera and OTUs with significance values is provided in Tables S5 to S8 below.

Table A: Excreta genera significantly altered by at-hatch administration at 14 days

Taxa	P value	Mean Ctrl	Mean Pb
**Unclassified Planococcaceae**	0.034	0.12	0.3
**Unclassified Clostridiales**	0.035	1.49	3.61
Lactococcus	0.038	0.16	0.07
Pseudoclavibacter	0.045	0	0.04
Nocardiopsis	0.045	0	0.1
Burkholderia	0.045	0	0.06
Brevundimonas	0.045	0	0.04

*The P-value was calculated using Wilcoxon test on square root transformed TSS normalised data. Mean values given for the control and probiotic group are also normalised and do not represent relative abundance.

Table B: Excreta OTUs significantly altered by at-hatch administration at 14 days

GreenGene annotation	OTUs by Qiime	Blast hit (16S Microbial NCBI)	% Id	P-value	Mean Ctr	Mean Pb1
**Firmicutes_g_*Clostridium_s_Clostridium bifermentans***	*40448*	*Clostridium irregulare*	83	0.0035	0	0.14
**Firmicutes_o_Clostridiales**	*31016*	*Turicibacter sanguinis*	91	0.0087	0	0.28
**Firmicutes_g_*Clostridium***	*58292*	*Clostridium chauvoei*	99	0.0087	0	0.37
**Firmicutes_g_*Lactobacillus***	*92949*	*Clostridium bartlettii*	89	0.012	0.05	0.22
**Firmicutes_g_*Clostridium***	*42048*	*Clostridium bartlettii*	89	0.02	0	0.04
**Firmicutes_g_*Ruminococcus***	*110830*	*Ruminococcus torques*	95	0.025	0.05	0
**Firmicutes_g_*Lactobacillus***	*990*	*Lactobacillus ingluviei*	88	0.025	0.14	0.4
**Firmicutes_g_*Coprococcus***	*103119*	*Ruminococcus lactaris*	94	0.026	0.15	0
**Proteobacteria_f_Enterobacteriaceae**	*56839*	*Escherichia fergusonii/Shigella sonnei*	89	0.033	0.31	0.15
**Firmicutes_g_*Lactobacillus***	*55066*	*Lactobacillus panis*	88	0.034	0.03	0.12
**Firmicutes_g_*Clostridium***	*78276*	*Blautia luti*	92	0.034	0.06	0.21
**Firmicutes_f_Planococcaceae**	*28277*	*Bacillus odysseyi*	100	0.034	0.12	0.3
**Firmicutes_f_Lachnospiraceae**	*3791*	*Clostridium citroniae*	87	0.036	0.19	0.08
**Firmicutes_g_*Lactococcus_s_Lactococcus lactis***	*65347*	*Lactococcus lactis*	98	0.038	0.16	0.07
**Firmicutes_o_Clostridiales**	*34656*	*Clostridium bartlettii*	91	0.043	0.03	0.1
**Firmicutes_o_Clostridiales**	*33821*	*Clostridium glycolicum*	97	0.043	1.14	3.33
**Firmicutes_g_*Lactobacillus***	*2757*	*Lactobacillus ingluviei*	99	0.043	1	2.34
**Proteobacteria_g_*Burkholderia_s_Burkholderia fungorum***	*7337*	*Burkholderia fungorum*	100	0.045	0	0.06
**Proteobacteria_g_*Brevundimonas***	*115675*	*Brevundimonas nasdae*	100	0.045	0	0.04
**Proteobacteria_f_Alcaligenaceae**	*95362*	*Parasutterella excrementihominis*	87	0.045	0	0.04
**Firmicutes_g_*Clostridium***	*61291*	*Clostridium lavalense*	92	0.045	0	0.05
**Firmicutes_g_*Blautia***	*20413*	*Blautia producta*	97	0.045	0	0.03
**Actinobacteria_g_*Pseudoclavibacter***	*14134*	*Curtobacterium flaccumfaciens*	98	0.045	0	0.04
**Actinobacteria_g_*Nocardiopsis***	*26604*	*Nocardiopsis alba*	100	0.045	0	0.1
**Firmicutes_g_*Paenibacillus***	*12572*	*Paenibacillus taiwanensis*	89	0.047	0.02	0.12
**Firmicutes_o_Clostridiales**	*4154*	*Clostridium glycolicum*	94	0.052	0.03	0.14

Table C: Excreta genera significantly altered by at-hatch administration at 28 days

Taxa	P value	Mean Ctrl	Mean Pb
Eubacterium	0.0013	0.58	0.34
Arthrobacter	0.018	0.06	0.01

Table D: Excreta OTUs significantly altered by at-hatch administration at 28 days

GreenGene annotation	OTUs by Qiime	Blast hit (16S Microbial NCBI)	% Id	P-value	Mean Ctr	Mean Pb1
**Firmicutes_f_Lachnospiraceae**	88432	*Ruminococcus lactaris*	96	0.00024	0.16	0.01
**Bacteroidetes_g_*Bacteroides_s_bacteroides uniformis***	71588	*Bacteroides uniformis*	100	0.00041	0	0.35
**Bacteroidetes_g_*Alistipes***	90591	*Alistipes onderdonkii*	100	0.00051	0.41	0.05
**Bacteroidetes_g_*Bacteroides_s_Bacteroides uniformis***	999	*Bacteroides uniformis*	97	0.0012	0	0.15
**Firmicutes_f_Lachnospiraceae**	31167	*Ruminococcus lactaris*	95	0.0019	0.34	0.13
**Bacteroidetes_g_*Alistipes***	13134	*Alistipes onderdonkii*	95	0.0019	0.2	0.04
**Firmicutes_f_Lachnospiraceae**	18967	*Ruminococcus lactaris*	96	0.0022	0.37	0.18
**Firmicutes_g_*Eubacterium***	82056	*Blautia schinkii*	95	0.0033	0.16	0.04
**Firmicutes_g_*Eubacterium***	65005	*Blautia luti*	97	0.0039	0.31	0.08
**Firmicutes_g_*Eubacterium***	860	*Blautia luti*	95	0.0043	0.19	0.07
**Firmicutes_f_Lachnospiraceae**	3791	*Clostridium citroniae*	87	0.0043	0.14	0.03
**Firmicutes_f_Lachnospiraceae**	76773	*Ruminococcus lactaris*	95	0.0047	0.29	0.15
**Firmicutes_f_Lachnospiraceae**	90852	*Ruminococcus lactaris*	95	0.011	0.18	0.08
**Firmicutes_g_Ruminococcus**	110830	*Ruminococcus torques*	95	0.012	0.19	0.1
**Firmicutes_f_Lachnospiraceae**	52684	*Ruminococcus lactaris*	94	0.012	0.25	0.15
**Firmicutes_o_Clostridiales**	34656	*Clostridium bartlettii*	91	0.018	0.07	0.01
**Firmicutes_g_Clostridium**	60034	*Clostridium citroniae*	92	0.018	0.78	0.56
**Firmicutes_f_Lachnospiraceae**	64017	*Clostridium bolteae*	95	0.018	0.16	0.07
**Actinobacteria_g_*Arthrobacter***	101679	*Clostridium bolteae*	95	0.018	0.06	0.01
**Firmicutes_f_Lachnospiraceae**	87732	*Clostridium hylemonae*	96	0.019	0.16	0.07
**Firmicutes_o_Clostridiales**	37819	*Clostridium cellulolyticum*	86	0.02	0.45	0.27
**Bacteroidetes_g_*Bacteroides_s_Bacteroidesuniformis***	13244	*Bacteroides uniformis*	96	0.02	0	0.09

**Bacteroidetes_o_Bacteroidales**	32789	*Eubacterium ruminantium*	86	0.021	0.2	0.1
**Firmicutes_f_Lachnospiraceae**	72951	*Lactonifactor longoviformis*	95	0.022	0.47	0.32
**Firmicutes_f_Lachnospiraceae**	54704	*Ruminococcus lactaris*	94	0.022	0.27	0.15
**Firmicutes_g_*Lactobacillus*_s_*Lactobacillusreuteri***	110349	*Lactobacillus reuteri*	100	0.024	0.42	0.03
**Firmicutes_f_Lachnospiraceae**	97957	*Ruminococcus lactaris*	97	0.024	0.22	0.12
**Firmicutes_f_Lachnospiraceae**	116114	*Clostridium phytofermentans*	92	0.024	0.23	0.12
**Firmicutes_g_Ruminococcus**	32851	*Blautia luti*	96	0.025	0.53	0.27
**Firmicutes_g_*Lactobacillus***	52248	*Lactobacillus ingluviei*	96	0.025	0.03	0
**Bacteroidetes_o_Bacteroidales**	16	*Clostridium irregulare*	87	0.025	0.12	0.05
**Firmicutes_o_Clostridiales**	22180	*Clostridium phytofermentans*	92	0.027	0.61	0.42
**Firmicutes_f_Lachnospiraceae**	80564	*Ruminococcus lactaris*	97	0.027	0.19	0.11
**Firmicutes_f_Lachnospiraceae**	69308	*Ruminococcus torques*	94	0.029	0.23	0.12
**Firmicutes_f_Lachnospiraceae**	58606	*Ruminococcus lactaris*	96	0.033	0.16	0.08
**Firmicutes_f_Lachnospiraceae**	93936	*Ruminococcus torques*	95	0.037	0.29	0.16
**Firmicutes_g_Clostridium**	64502	*Ruminococcus lactaris*	97	0.038	0.16	0.08
**Firmicutes_g_*Eubacterium***	80436	*Blautia luti*	94	0.04	0.15	0.06
**Firmicutes_g_*Clostridium***	81771	*Clostridium lavalense*	93	0.044	0.22	0.15
**Firmicutes_f_Lachnospiraceae**	10489	*Ruminococcus lactaris*	93	0.044	0.16	0.07
**Firmicutes_g_Ruminococcus**	88419	*Ruminococcus torques*	95	0.045	0.2	0.13
**Firmicutes_g_*Clostridium***	78276	*Blautia luti*	92	0.045	0.36	0.27
**Firmicutes_o_Clostridiales**	78661	*Shigella dysenteriae*	88	0.047	0.09	0.04
**Firmicutes_g_*Lactobacillus***	37949	*Lactobacillus reuteri*	87	0.047	0.18	0.04
**Firmicutes_g_*Ruminococcus***	845	*Clostridium xylanolyticum*	96	0.05	0.26	0.17
**Firmicutes_f_Lachnospiraceae**	32964	*Ruminococcus torques*	95	0.05	0.79	0.61

Table E: Caecal genera significantly altered by at-hatch administration at 28 days

Taxa	P value	Mean Ctrl	Mean Pb
**Brachybacterium**	0.0056	0.39	0.19
**Arthrobacter**	0.017	0.04	0
**Eubacterium**	0.034	0.57	0.47
**Coprobacillus**	0.036	0.52	0.7
**Alistipes**	0.045	1.02	0.63

Table F: Caecal OTUs significantly altered by at-hatch administration at 28 days

GreenGene annotation	OTUs by Qiime	Blast hit (16S Microbial NCBI)	% Id	P-value	Mean Ctr	Mean Pb1
**Bacteroidetes_g_*Bacteroides_s_bacteroides uniformis***	71588	*Bacteroides uniformis*	100	0.00024	0	0.99
**Bacteroidetes_g_*Bacteroides_s_bacteroides uniformis***	13244	*Bacteroides uniformis*	96	0.00024	0	0.34
**Bacteroidetes_g_*Bacteroides_s_bacteroides uniformis***	999	*Bacteroides uniformis*	97	0.00034	0.02	0.45
**Bacteroidetes_g_*Alistipes***	90591	*Alistipes onderdonkii*	100	0.00069	0.76	0.07
**Bacteroidetes_g_*Alistipes***	13134	*Alistipes onderdonkii*	95	0.0047	0.33	0.07
**Firmicutes_g_*Ruminococcus***	71066	*Ruminococcus torques*	96	0.0049	0.33	0.46
**Actinobacteria_g_*Brachybacterium***	64941	*Brachybacterium faecium*	100	0.0056	0.39	0.19
**Firmicutes_f_Lachnospiraceae**	31167	*Ruminococcus lactaris*	95	0.0058	0.42	0.28
**Firmicutes_f_Lachnospiraceae**	42809	*Clostridium saccharolyticum*	96	0.0067	0.37	0.52
**Firmicutes_g_*Ruminococcus***	46597	*Ruminococcus torques*	95	0.0087	0.47	0.63
**Firmicutes_o_Clostridiales**	16691	*Clostridium populeti*	93	0.013	0.32	0.11
**Firmicutes_g_*Eubacterium***	65005	*Blautia luti strain*	97	0.014	0.32	0.18
**Tenericutes_g_Coprobacillus**	111142	*Clostridium xylanolyticum*	85	0.016	0.18	0.26
**Firmicutes_f_Lachnospiraceae**	62279	*Clostridium hylemonae*	94	0.016	0.14	0.19
**Actinobacteria_g_*Arthrobacter***	101679	*Arthrobacter protophormiae*	100	0.017	0.04	0
**Firmicutes_f_Lachnospiraceae**	81021	*Clostridium* s. N6	94	0.021	0.3	0.41
**Firmicutes_f_Lachnospiraceae**	29697	*Clostridium hylemonae*	97	0.026	0.46	0.58
**Firmicutes_f_Lachnospiraceae**	81590	*Ruminococcus lactaris*	96	0.028	0.09	0.27
**Firmicutes_g_*Clostridium***	22207	*Clostridium saccharolyticum*	96	0.032	0.17	0.22
**Firmicutes_f_Lachnospiraceae**	88432	*Ruminococcus lactaris*	96	0.032	0.18	0.09

GreenGene annotation	OTUs by Qiime	Blast hit (16S Microbial NCBI)	% Id	P-value	Mean Ctr	Mean Pb1
**Firmicutes_f_Lachnospiraceae**	60054	*Ruminococcus lactaris*	97	0.032	0.25	0.33
**Actinobacteria_g_*Adlercreutzia***	83905	*Adlercreutzia equolifaciens*	96	0.032	0.14	0.2
**Tenericutes_g_*Coprobacillus***	38777	*Clostridium cocleatum*	91	0.033	0.42	0.55
**Actinobacteria_g_*Adlercreutzia***	45835	*Adlercreutzia equolifaciens*	99	0.033	0.17	0.25
**Bacteroidetes_o_Bacteroidales**	16	*Clostridium irregulare s.6VI*	87	0.036	0.21	0.13
**Firmicutes_g_*Blautia***	77309	*Ruminococcus lactaris*	97	0.041	0.36	0.8
**Firmicutes_f_Lachnospiraceae**	29757	*Ruminococcus lactaris*	96	0.041	0.24	0.46
**Actinobacteria_f_Dermabacteraceae**	67140	*Brachybacterium rhamnosum*	97	0.041	0.25	0.12
**Firmicutes_f_Lachnospiraceae**	62881	*Clostridium hylemonae s.TN-272*	97	0.043	1.38	1.77
**Firmicutes_f_Lachnospiraceae**	62791	*Clostridium hylemonae s.TN-272*	96	0.043	0.22	0.29
**Firmicutes_g_Turicibacter**	93943	*Clostridium populeti*	89	0.045	0.33	0.18
**Firmicutes_g_*Lactobacillus_s_lactobacillus reuteri***	79887	*Lactobacillus reuteri*	97	0.047	0.05	0
**Firmicutes_g_*Turicibacter***	101382	*Clostridium haemolyticum s.ATCC9650*	89	0.048	0.06	0.02
**Firmicutes_f_Lachnospiraceae**	33535	*Ruminococcus lactaris*	93	0.049	0.18	0.1
**Tenericutes_g_*Coprobacillus***	114607	*Clostridium cocleatum*	89	0.05	0.17	0.27

Table G: Ileum genera significantly altered by at-hatch administration at 28 days

Taxa	P value	Mean Ctrl	Mean Pb
Weissella	0.006	0.03	0.42
Paracoccus	0.025	0.09	0.68
**Unclassified Clostridiales**	0.031	3.46	2.03

Table H: Ileum OTUs significantly altered by at-hatch administration at 28 days

GreenGene annotation	OTUs by Qiime	Blast hit (16S Microbial NCBI)	% Id	P-value	Mean Ctr	Mean Pb1
**Proteobacteria_f_Enterobacteriaceae**	18699	*Shigella dysenteriae*	97	0.0023	0.08	0
**Firmicutes_f_Lachnospiraceae**	74903	*Turicibacter sanguinis*	87	0.0036	0.1	0.02
**Firmicutes_g_*Clostridium***	49919	*Clostridium glycolicum*	99	0.006	0.27	0.04
**Firmicutes_g_*Turicibacter***	52163	*Turicibacter sanguinis*	89	0.006	0.12	0.01
**Firmicutes_g_*Weissella***	35028	*Weissella paramesenteroides*	100	0.006	0.03	0.42
**Firmicutes_g_*Staphylococcus***	30640	*Staphylococcus cohnii*	97	0.018	0.01	0.13
**Firmicutes_f_Ruminococcaceae**	78501	*Clostridium sporosphaeroides*	91	0.019	0.04	0
**Firmicutes_g_*Lactobacillus*_s_*lactobacillushelveticus***	56056	*Lactobacillus kitasatonis*	96	0.019	0.15	0
**Firmicutes_o_Clostridiales**	111588	*Clostridium glycolicum*	95	0.019	0.16	0.05
**Firmicutes_g_*Lactobacillus*_s_*lactobacillushelveticus***	46075	*Lactobacillus crispatus*	96	0.02	0.09	0.02
**Firmicutes_o_Clostridiales**	31016	*Turicibacter sanguinis*	91	0.02	0.12	0.02
**Firmicutes_o_Clostridiales**	4154	*Clostridium glycolicum*	94	0.02	0.15	0.05
**Firmicutes_o_Clostridiales**	73173	*Clostridium glycolicum*	94	0.023	0.2	0.06
**Proteobacteria_g_*Paracoccus***	49975	*Paracoccus aminovorans*	99	0.025	0.09	0.68
**Firmicutes_f_Ruminococcaceae**	40813	*Bacteroides capillosus*	95	0.031	0.09	0.01
**Firmicutes_g_*Lactobacillus***	115894	*Lactobacillus kitasatonis*	89	0.031	0.08	0.01
**Firmicutes_g_*Lactobacillus*_s_*lactobacillushelveticus***	28599	*Lactobacillus kitasatonis*	96	0.039	0.09	0.01
**Firmicutes_f_Aerococcaceae**	21216	*Globicatella sulfidifaciens*	99	0.04	0.54	0.19
**Firmicutes_o_Clostridiales**	33821	*Clostridium glycolicum*	97	0.046	3.29	1.91
**Firmicutes_f_Ruminococcaceae**	83533	*Faecalibacterium prausnitzii*	91	0.049	0.06	0.01
**Firmicutes_f_Lachnospiraceae**	16652	*Lactobacillus reuteri*	87	0.05	0.06	0
**Firmicutes_f_Lachnospiraceae**	44609	*Ruminococcus torques*	96	0.05	0.02	0
**Firmicutes_f_Lachnospiraceae**	81021	*Clostridium s.N6*	94	0.05	0.02	0
**Firmicutes_g_*Clostridium***	81771	*Clostridium lavalense*	93	0.05	0.02	0
